# Supplementary material for: Energy requirements for securing wellbeing in Switzerland and the space for affluence and inequality
Source: Nat Commun. 2025 Apr 30;16:4066. doi: 10.1038/s41467-025-59276-2 (PMC12043928; doi:10.1038/s41467-025-59276-2)
Supplement: Supplementary file 1 — Supplementary Information [file 41467_2025_59276_MOESM1_ESM.pdf]

Supplementary Information

Energy requirements for securing wellbeing in Switzerland and the space for affluence and inequality

Joel Millward-Hopkins[1], Vivien Fisch-Romito[1], Sascha Nick, and Emile Chevrel*

[1]University of Lausanne (UNIL), Lausanne, Switzerland

*Corresponding author, joeltmh@gmail.com

Contents

Supplementary Methods 1: Quantifying DLS for Switzerland 1

Nutrition 2

Shelter & living conditions 3

Hygiene 3

Clothing 3

Healthcare and Education 3

Communication & information 4

Mobility 4

Public space 8

Public administration 8

Unspecified sectors 8

Supplementary Methods 2: Energy intensities 9

Projecting energy intensity reductions 9

Supplementary References 12

Display items:

Supplementary Figure 1 5

Supplementary Table 1 2

Supplementary Table 2 6

Supplementary Table 3 6

Supplementary Table 4 7

Supplementary Table 5 7

Supplementary Table 6 10

Supplementary Methods 1: Quantifying DLS for Switzerland

Below we describe the values used for DLS to produce our DLE estimate of Switzerland. However, note that while most previous DLE applies a fully bottom-up approach, estimating a DLS activity-level and associated energy intensity for each item in the DLS inventory (e.g., a required pkm/cap/year for transport, and an associated MJ/pkm), in this work we sometimes take a different approach. This is to allow the use of energy scenario data specific to Switzerland, which does not align cleanly with the DLS categories. We describe our approach to this in the energy section below.

Nutrition

To specify the food required in Switzerland (per person, on average), we look to data from the Food and Agriculture Organization of the United Nations[1]. This includes data on the 'Average dietary energy requirement' for Switzerland, described as 'a proper normative reference for adequate nutrition in the population'. Alongside this, they report the 'Dietary energy supply', which, when divided by the requirement, gives the 'Average Dietary Energy Supply Adequacy (ADESA)'.

Data for Switzerland is included in Supplementary Table 1 below. For the DLS activity-level, we assume that the FAO's dietary energy requirement estimate is appropriate, which means the ADESA is essentially a measure of overconsumption beyond DLS. We take the 3-year average of **2,557 kcal/cap/day** (2020-2022), although ADESA in Switzerland appears stable. Note that Supplementary Table 1 also includes for comparison the FAO's 'Minimum dietary energy requirement' for Switzerland, which represents average energy requirements to maintain health assuming low levels of physical activity.

We also consider dietary change as an aspect of DLS. Specifically, we look at consumption of all animal-based foods as a fraction of total kcal, taking the DLS level as the **12.1%** suggested in the *Eat Lancet* report[2]. This compares to current Swiss consumption of 26-27% reported by the FAO[1] (Supplementary Table 1). This approach is simpler than in previous global DLE work, which used more disaggregate diets, but this was necessary for the energy calculations described later.

The final aspect of the nutrition sector involves appliances for cooking and cold storage. Previous work considered these in a fully bottom-up manner, looking to Life Cycle Assessment studies of individual appliances. In the current work, however, our approach to energy intensities only allowed for the consideration of an aggregate 'consumer goods' category, within which cooking and cold storage appliances are accounted for. Regarding ownership, our assumptions are intended to be consistent with continued ownership of such appliances within each household.

Supplementary Table 1: Food supply and food requirements in Switzerland
Data form the FAO[1] describing various measures of food consumption and requirements for Switzerland. Average Dietary Energy Supply Adequacy is only officially reported by the FAO in 3- year averages, so the values shown here are our yearly calculations. The final row shows the Eat Lancet[2] recommendation for the consumption of all animal-products, calculated on an energy basis (i.e. recommended consumption of animal-products in kcal, divided by total recommended consumption of kcal; this does not vary over time).

Measure	2020	2021	2022	Units
Minimum dietary energy requirement	1,959	1,958	1,957	kcal/cap/day
Average dietary energy requirement	2,558	2,557	2,555	kcal/cap/day
Dietary energy supply	3,410	3,452	3,402	kcal/cap/day
Average Dietary Energy Supply Adequacy	133	135	133	%
Total animal-based energy supply	26.3	27.0	-	%
Eat Lancet recommendation; all animal-foods	12.1	12.1	12.1	%

Shelter & living conditions

The key DLS activity-level for this category is residential floor-space. In previous global DLE work[3], the floorspace of a household was assumed to be 10 m^2 of private per person and 20 m^2 of shared space per house, which, with a household size of four people, lead to an average of 15 m^2/cap. We use the same base assumptions here, but with the Swiss average household size of ~2, which leads to the slightly larger **DLS floorspace of 20 m^2/cap**. As described in the energy section, this floorspace is used to calculate energy requirements for all subcategories – thermal comfort, lighting, and household furnishing.

This 20 m^2/cap assumption remains well under the Swiss average of ~47 m^2/cap, but it is reasonably close to the 28 m^2/cap living space typical of foreign-resident households in Geneva[4,5] (the Canton with the smallest average living space), or the 10% of three-person Swiss households with the smallest living space (~25 m^2/cap).

Hygiene

DLS levels of residential water use, and hot water use within this, are taken from previous DLE work where they were assumed to be **50 and 20 L/cap/day**, respectively[3]. These assumptions were themselves based upon other studies of the water requirements of meeting basic human needs[6], although our assumption is toward the lower end of the UN/WHO recommendation of 50-100 L/cap/day[7]. It is, of course, also much lower than current average Swiss water consumption, which is ~160 L/cap/day[8].

Cleaning products were absent from previous DLS and DLE work, and it is extremely difficult to make specific bottom-up assumptions (i.e., exactly how much and what personal hygiene and household cleaning products are sufficient for a person to use annually). We also found no appropriate sufficiency work in the literature. Consequently, we use a rough, heuristic downscaling factor. This assumes that current consumption of cleaning products overshoots a DLS level by that same magnitude that the total Swiss material footprint currently overshoots DLS material footprint estimate. Specifically, Eurostat report Swiss *raw material consumption* at 15.2 T/cap in 2022[9], while Vélez-Henao and Pauliuk[10] estimate that DLE requires a material footprint of around 3 T/cap in a 'low' material intensity scenario assuming dietary change, increased active mobility, etc. This implies overconsumption of a factor of ~5, and hence our DLS assumption for cleaning product is simply an **80% reduction** on today's levels. We reuse this heuristic downscaling factor for other sectors, including the final subsection of hygiene, namely waste management.

Clothing

DLS levels of clothing consumption are taken from previous global DLE work[3], as there is no obvious reason Swiss basic needs for clothing differ from the generic assumptions made there. Specifically, the DLS value for new clothing (including other household textiles) is assumed to be **3.6 kg/cap/year**. Clothes washing appliances are treated as cooking and cold storage were, and aggregated into the 'consumer goods' category described in the energy section. Again, the assumption is intended to be consistent with continued ownership at the household and communal levels.

Healthcare and Education

For healthcare, previous DLE global work assumed a minimum floor area of hospitals is required per person for DLS, then estimated the direct and wider energy usage of this. That approach was crude, however, and here we take an equally simply approach, but one grounded in health outcomes.

We look to recent work analysing energy footprints of healthcare systems in 49 countries and world regions, then compare the Swiss footprint to other 'best practice' countries with equally high health outcomes. Switzerland is reported to have a footprint of over 15 GJ/cap/year in 2015 (the second highest in the study), which is largely indirect energy use. Other countries scoring similarly (~90) on the *Healthcare Access and Quality* index that the study uses have footprints as low as 3.5, 4.7, and 5.7 GJ (in Spain, Italy, and Sweden, respectively). We therefore take the average of these values (4.6 GJ) as best practice and assume the healthcare system of Switzerland could achieve similarly high outcomes while using 30% (4.6/15.3) of its current energy. Note that the simplicity of this approach means the DLS estimate is inseparable from the energy intensity estimated.

For education, we follow previous DLE work, which assumed educational facilities are sized to offer 10 m^2/pupil, and then obtains a total floor space requirement by multiplying this 10 m^2 with the population that is in the main educational age bracket. Here, we take this age range as 5-21 and in 2020, 1.7 million of Switzerland's 8.6 million population (20%) were of this age. By 2050, this fraction drops slightly to 18%. Overall, the average floor space of educational buildings drops from **2 m^2/cap** to **1.8 m^2/cap** from 2020-2050, significantly less than the current value of 3.3 m^2/cap.

Communication & information

Previous global DLE work considered communication & information in a fully bottom-up way, thus estimating the energy use of producing and using phones, laptops, and supporting network infrastructure. Here we use a simple ratio to downscale Swiss household use of information technology to an assumed DLS level. To estimate this (assumed) ratio, we look at current internet use-patterns in Switzerland and assess what fraction of current use can be considered as contributing to basic needs.

Data has been reported for Switzerland that describes the average amount of time spent daily on different uses of media[11]. This suggests that Swiss internet users spend around 5 hrs 40 mins online each day, with educational/research accounting for ~25% of this and social media ~15%. These are the two key aspects of internet use that contribute to basic needs (arguably, of course), while uses like music streaming and gaming can be considered outside the scope of DLS. We thus assume that **one third of current Swiss internet** use is sufficient for DLS, by taking the educational use (25%) and half of the social media use (~8%; thus assuming that social media is overused by a factor of two).

This is a rough estimate, but it can be broadly justified further by the same report. Swiss internet users' self-reported main reasons for using the internet are "finding information", "keeping up to date with news and events", "researching how to do things", and "staying in touch with family and friends" – the main reasons people say they need to use the internet are thus *educational and social*, so well-aligned with basic needs. However, data from the report shows that the websites most visited by Swiss internet users are not well aligned with these uses – for example, at least 4 of the 20 most visited sites are pornography.

Mobility

Much of the existing DLS literature is crude in its estimates of mobility needs[10,12,13]. Here, in contrast, mobility is the sector we consider in the most detail when estimating DLS activity levels, due to both its significance in energy terms and the influence of geographic specificities on the necessary mobility. We estimate annual passenger kilometres required in Switzerland to meet basic needs, and how these may be distributed across modes. To do this we consider three 'variables' (which is this case are not single numbers), beginning with current empirical data, before applying normative assumptions about how much of current travel can be considered part of decent living (see Supplementary Figure 1).

The first variable is the number of trips that are currently taken by Swiss residents for different purposes. We use data from the Swiss Microcensus[14] that summarises the average numbers of trips taken by people for 7 different purposes – *Work*; *Training, school*; *Shopping*; *Leisure*; *Work & service travel*; *Services & accompanying others*; *Other*. We consider data for three different age groups, namely those of *pre-working age* (<18), those of *working age*, and those of *post-working age* (64<), as these have very different compositions of trips (for obvious reasons). The number of trips Swiss residents take is left largely unchanged in the DLS estimate, aside from two modifications. First, we reduce the number of trips taken for shopping down to an average of 1.25/cap/week (for all age ranges), based upon the normative assumption that one trip a week plus an additional more specialised trip every 4 weeks is sufficient for decent living. This represents a ~50% reduction on current levels. Second, we reduce the number of work trips by ~20%, based on the assumption[15] that the hours worked by full-time employees reduces to 32/week (down from the current 40) and the share of employees working part-time continues to increase as it has over the past three decades (we assume it reaches 47%, up from 38% today[16,17]). We are thus assuming that inherent to decent living is shorter working weeks (and that work travel is linearly related to hours worked).

The second variable is the location of the facilities and services that serve the purposes of people's trips. Here, we build on the zonal methodology of Kompil et al.[18]. They propose a typology of *local*, *subregional*, and *regional* service areas – each of which contains various services – and they estimate the average size of these service areas in European countries (Supplementary Table 2). Using accessibility data for 30 types of services in Switzerland for *Urban*, *Intermediate* & *Rural* geographies[19], we estimate distances to the closest local, subregional, and regional facilities defined by Kompil et al.[18] (see Supplementary Table 3). We then add an *Extra-regional* zone to allow consideration of lengthier trips to friends and relatives, setting this to a round ~100 km. The biggest difficulty lies in defining, for each purpose, within what zones people's trips typically take place. For some purposes this is easy – *Training, school* can be spread evenly across local and subregional zones, simply due to the zones' definitions; *Shopping* can be split 80/20 due to the abovementioned

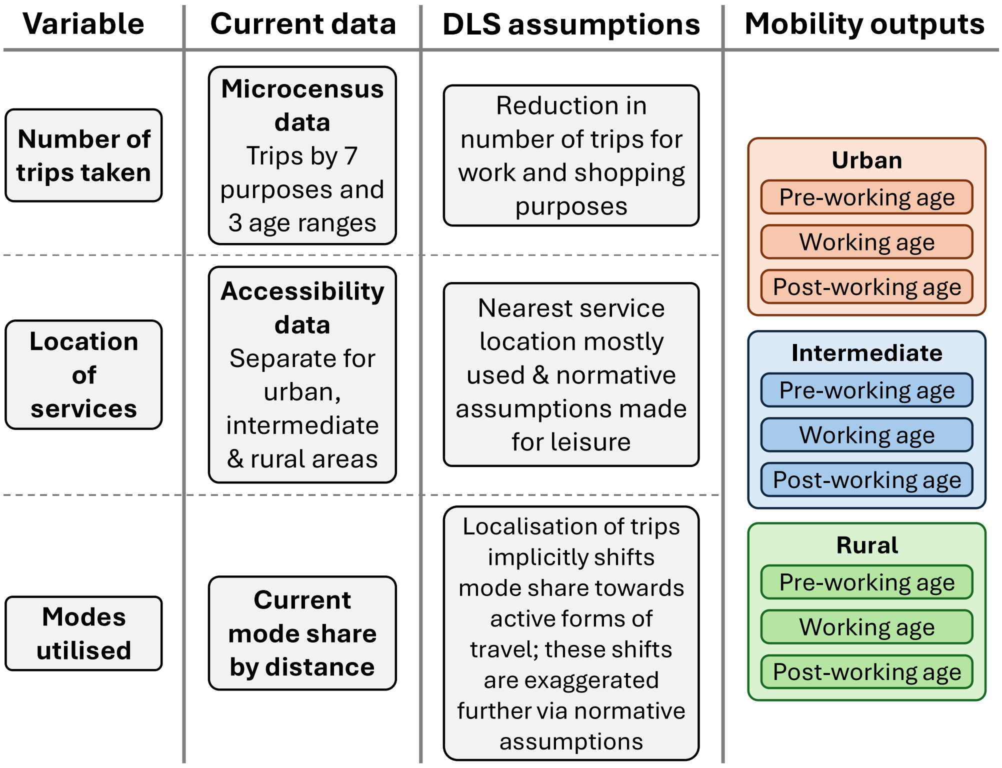

Supplementary Figure 1: Current methodology for estimating DLS mobility
Overview of DLS mobility methodology, including variables, data sources, assumptions, and outputs.

Supplementary Table 2: Description and definition of Kompil's[18] zonal methodology

Zone definitions	Ideal service area population	Ideal service area distance	Estimated distance to access nearest facilities in the EU28	
			Cities	Rural areas
**Local (neighbourhood) facilities**<br>Schools, small health facilities, childcare services, sport facilities, small markets etc.	10,000	2.5 km	2 km	9 km
**Subregional (municipal) facilities**<br>High schools, hospitals, theatres, cultural facilities, supermarkets, hobby markets etc.	100,000	10 km	4 km	18 km
**Regional facilities**<br>Specialized education & health centres, large sports & cultural facilities, governmental organizations, other high-tech services etc.	1,000,000	50 km	12 km	48 km

assumption of one trip a week plus an additional (longer distance) trip per 4 weeks. But the imperative of DLS – to define the minimum consumption necessary to meet human needs – is not easy to apply to other categories, particularly the significant purposes of *Work* and *Leisure*. For the former, we simply assume an even split across local, subregional, and regional zones, as cohabitation of couples, relatives, and friends may necessitate longer trips for work by some household members. For leisure, we assume one trip every two weeks is regional, and one every eight weeks is extra-regional, with the remaining trips evenly split between local and subregional zones. These are perhaps the most arbitrary and significant assumptions in our DLE model and would benefit from revision in future work.

The third variable is the mode share and how this varies with both zone and geography. Microsensus data shows how the mode share of transport in Switzerland varies with trip distance, for both work and leisure[20]. The work-travel mode share of walking & cycling, for example, varies from ~90% for distances under 0.5 km, to ~4% for distances of 10 km, while the mode share of cars increases from <10% to ~70% over the same distance. We use this data to estimate the mode share within each zone of each geography (Supplementary Table 4). Two aspects of our assumptions shift the mode shares away from private vehicles and towards active travel and public transport, consistent with the DLS imperative to reduce the material consumption required to meet human needs: First, the zonal assumption described above serves to localise transport, which implicitly shifts transport towards walking and cycling, which are far more commonly used in Switzerland for shorter trips. Second, we generally round the mode-share data to the nearest 5% or 10%, rounding

Supplementary Table 3: Service zones in Switzerland and assumed purpose locations
Estimated size of Swiss services zones – i.e., average distance to access the nearest services in each of the three geographies – and our assumed distributions of purposes within these four zones.

Zones	Accessibility of nearest service in Switzerland			Assumed locations of purposes						
	Urban	Inter-mediate	Rural	Work	Training, school	Shopping	Leisure	Work & service travel	Services & accom others	Other
**Local**	1 km	2 km	3 km	33%	50%	80%	44%	-	50%	33%
**Subregional**	3 km	7 km	10 km	33%	50%	20%	44%	50%	50%	33%
**Regional**	4 km	10 km	15 km	33%	-	-	10%	50%	-	33%
**Extra-regional**	100 km			-	-	-	2.5%	-	-	-

Supplementary Table 4: Assumed shares of transport modes by zone and geography

Mode of transport	Urban				Intermediate				Rural			
	Local	*Subregional	Regional	⁺Extra-regional	Local	Subregional	ªRegional	⁺Extra-regional	*Local	ªSubregional	Regional	⁺Extra-regional
**Walking & cycling**	80%	25%	20%	0%	50%	15%	10%	0%	25%	10%	5%	0%
**Cars & motorbikes**	10%	50%	55%	50%	35%	60%	60%	50%	50%	60%	65%	50%
**Buses**	10%	13%	13%	0%	10%	13%	15%	0%	13%	15%	15%	0%
**Rail**	0%	13%	13%	50%	5%	13%	15%	50%	13%	15%	15%	50%

the share of cars down and that of public transport and active travel up. This represents another normative assumption, albeit a very conservate one compared to previous DLE work, which assumed mode shares of car transport fall to well under 20% in 2050[3].

Once these various calculations and assumptions have been made, the resulting DLS mobility levels are easily obtained. To obtain total pkm/capita, one simply multiplies the number of trips for each purpose (which varies across age groups, but not geographies), by the zonal location of these trips from Supplementary Table 3 (which varies across purposes, but not geographies or age groups) to get total trips per zone, and then by the average travel distances for each zone from Supplementary Table 3 (which vary across geographies only). Then, to obtain pkm/capita for each mode, one multiplies these totals by the mode shares of Supplementary Table 4. Finally, we add a fixed amount of air travel for each person, using the global average value (550 pkm/cap/yr) assumed in the Low Energy Demand scenario of Grubler et al.[21], which is consistent with a 1.5°C future of low energy demand ad low reliance on negative emissions technologies. Results are shown in Supplementary Table 5 aggregated across age groups (with the 2025 Swiss population), where they are also compared to the values assumed for Switzerland in previous global DLE work. The total travel assumed is almost identical, however, the current work assumes much greater car travel, and significantly less public transport and active travel.

Supplementary Table 5: Assumed DLS mobility levels in Switzerland
Values are for 2025 and in pkm/cap/year. For comparison, assumptions from Millward-Hopkins et al.[3] are shown in the rightmost column.

Mode of transport	Urban	Intermediate	Rural	Switzerland	MH 2020
**Walking & cycling**	789	1,033	863	854	1,251
**Cars & motorbikes**	1,538	3,428	5,034	2,464	716
**Buses**	267	650	1,088	471	1,431
**Rail**	806	1,189	1,674	1,017	1,431
**Air transport**	550	550	550	550	1,000
**Total**	**3,950**	**6,850**	**9,208**	**5,356**	**5,829**

Public space

Public indoor space was absent from previous DLE work, despite public space as a place to exercise "freedom to gather/dissent" being included in the original DLS framework – albeit without a estimation of how much space may be required[22]. We include indoor public space in the Swiss DLE estimate – spaces that could host leisure, art, culture, or general community gathering – and omit outdoor spaces, which likely use negligible amounts of energy. No specific guidelines were found in the literature that could be input into the DLE model, so we make a rough estimate. This begins by assuming that public spaces provide every person attending with 10 m^2 of space on average, which is the DLS estimate for personal household space[22]. We then assume citizens require access to two different types of public space per week and another type of space once a month, then assume (conservatively) that public spaces can host two different groups per day. This means that total public space must be sufficient to provide, on average, **floor space of 1.6 m^2/capita**, i.e., 10 x (2/(7x2) + 1/(30x2)).

These assumptions are arbitrary, but they seem reasonable and other assumptions lead to similar estimates – for example, assuming people need to access 3-4 public spaces per week and that spaces can serve 3-4 groups a day implies a floor space of ~1.4 m^2/capita; assuming access to 2 spaces per week that serve 3 groups a day, but doubling the space requirement to 20 m^2, implies a floor space of ~1.9 m^2/capita. Further, the floor space of miscellaneous services in Switzerland is currently 3.7 m^2/capita, which includes DLS-related buildings such as arts, recreational, cultural, and community buildings, but also those relating to legal, real estate, and other administrations (see Energy Perspectives 2050+). It seems a reasonable first estimate for the DLS level of public space to be around half of this (in any case, the sector contributes little energy to total DLE, so uncertainties here are of minor consequence).

Public administration

The original DLS framework, and subsequent DLE work, did not consider the higher-level public administration that may be required to organise the provision of the public services within DLS. It also did not consider other activities that are (arguably) necessary for social stability, most notably in the current geopolitical climate, national security. We thus add *public administration* to our current DLE estimate, as this is a sector in the energy scenarios we use (and it includes defence activities). We do not estimate a DLS level but just assume that current public administration in Switzerland is reasonable, as Switzerland ranks 2nd highest, globally, on the World Bank Government Effectiveness indicator[23]. This is likely an overestimate, but even so, it accounts for only ~4% of total DLE.

Unspecified sectors

Following previous global work, the final part of estimating DLE involves adding supporting activities – retail services, freight transport, and domestic electricity infrastructure. For retail and freight, we use the same heuristic downscaling factor as we applied to cleaning products, namely, the ratio of the current Swiss material footprint to a DLS material footprint estimate. We thus reduce Swiss retail floorspace and domestic freight transport by 80%, which gives **1 m^2/capita of floor space** and **~660 tkm/capita of freight** (400 by road, 260 by rail; see Energy Perspectives 2050+). Note that the way we calculate DLE energy intensities means that international freight is accounted for indirectly but not quantified exactly in tkm. However, energy footprint data suggests that the total freight-related energy use embodied in Swiss consumption is nearly 3 times larger than the domestic fraction alone (see Energy Intensities section), so our implicit DLS estimate is closer to 2,000 tkm/capita. This is very similar to previous global work, which made a crude estimate of 2,300 tkm/capita of freight for DLS[3]. For domestic electricity infrastructure there is no DLS level – the required infrastructure is simply determined by the amount of domestic electricity needed to provide the DLS inventory.

Supplementary Methods 2: Energy intensities

Projecting energy intensity reductions

EP2050+ offers energy intensities from 2020-2050 for domestic sectors, and hence it captures the deployment of more efficient technologies. However, estimates must also be made of how the energy intensities related to the imported, embodied energy that completes the Swiss energy footprint change over the same time horizon. Doing this thoroughly is an enormous task, so we primarily use two simplified assumptions:

First, where EXIOBASE is used to scale up a domestic EP2050+ value, we use the same factor throughout the time horizon. Essentially, this means we assume that sectoral energy efficiency improvements occurring in Switzerland are mirrored in global supply chains.

Second, where absolute values from EXIOBASE are used, we assume energy intensities improve at the global rates estimated by the IEA's *Net Zero by 2050* scenario[24], using improvements from the closest high-level industrial sectors. These matches are not perfect, however, as the IEA sectors are broad. For clothing there is no appropriate match, so we use the energy intensity improvements from recent circular economy work[25].

Supplementary Table 6 below summarises all the energy intensities used.

Supplementary Table 6: Summary of energy intensities used in the DLE model and their sources

In the 'Data' column, *EP* indicates that the data source is EP2050+; *EX-HH* and *EX-Tot* that the data is from EXIOBASE (using only household demand and total demand, respectively); *EP-EX* indicates that EP2050+ data is scaled up to include imported energy using EXIOBASE; and *Other* indicates that another data source is used, as detailed in the 'Notes' column. Where energy intensity projections are not from EP2050+, the source is also detailed in the 'Notes' column (Hertwich et al.[26]; Hoxha & Jusselme[27]; Millward-Hopkins et al.[25]; Panos et al. [28]). Primarily, the source here is the IEA's Net Zero scenario[24].

Category	Sub-category	Data	2020	2025	2030	2035	2040	2045	2050	Units	Notes
Domestic energy											
Residential	Space heating	EP	304	260	215	177	145	119	98	MJ/m^2	-
	Cooling	EP	7.3	7.1	7.3	7.9	8.8	9.4	10.1	MJ/m^2	-
	Lighting	EP	7.4	4.7	4.0	3.6	3.3	3.0	2.8	MJ/m^2	-
	Appliances	EP	1,159	1,037	940	885	854	826	798	MJ/cap	-
	Information technology	EP	672	674	670	659	638	615	594	MJ/cap	-
	Water heating	EP	3,419	3,047	2,658	2,311	2,031	1,791	1,584	MJ/cap	-
	Cooking	EP	655	634	608	582	559	544	539	MJ/cap	-
	Clothing	EX-HH	39	37	35	33	32	30	29	MJ/cap	Projection from Millward-Hopkins et al. (2023)
	Cleaning products*	EX-HH	2.2	2.1	2.1	2.0	1.9	1.9	1.9	MJ/cap	Projection from WEO NZ2050, industry - chemicals
	Household furnishings*		0.010	0.009	0.009	0.009	0.008	0.008	0.007	MJ/m^2	Projection from WEO NZ2050, industry - all<br>Energy intensity from Hoxha & Jusselme (2017)
	Other consumer goods	EX-HH	156	149	142	136	129	123	117	MJ/cap	Projection from WEO NZ2050, industry - all
Transport	Cars and motorbikes	EP	1.08	0.98	0.84	0.71	0.60	0.52	0.46	MJ/pkm	-
	Buses		0.18	0.17	0.15	0.15	0.14	0.13	0.13	MJ/pkm	Energy intensities from Panos et al. (2023), ICE & electric buses
	Rail	EP	0.29	0.27	0.26	0.24	0.23	0.22	0.22	MJ/pkm	-
	Air transport	EP	1.18	1.07	0.96	0.87	0.78	0.71	0.64	MJ/pkm	-
	Road transport (beyond direct)	EX-Tot	0.14	0.13	0.12	0.11	0.09	0.08	0.06	MJ/pkm	Projection from WEO NZ2050, industry - metals
	Rail transport (beyond direct)	EX-Tot	0.32	0.29	0.27	0.24	0.20	0.17	0.14	MJ/pkm	Projection from WEO NZ2050, industry - metals
	Air transport (beyond direct)	EX-Tot	0.30	0.29	0.27	0.26	0.24	0.23	0.21	MJ/pkm	Projection from WEO NZ2050, industry - metals & concrete
Services	Healthcare	EP	1,895	1,666	1,509	1,410	1,329	1,259	1,217	MJ/cap	-
	Education	EP	534	464	414	374	334	309	290	MJ/m^2	-
	Public administration	EP	665	570	519	465	412	382	356	MJ/cap	-
	Trade	EP	631	564	507	457	407	371	345	MJ/m^2	-
	Telecommunication	EP	1,513	1,326	1,180	1,063	961	887	817	MJ/cap	-
	Public space*	EP	582	514	461	416	370	340	318	MJ/m^2	Average of Education, Public administration & Trade sectors above

**Industry**	Water supply & waste management	EP	184	136	111	95	83	75	68	MJ/cap	-
	Construction	EP	671	638	606	579	558	538	519	MJ/cap	-
	Electricity infrastructure		0.17	0.16	0.16	0.15	0.14	0.13	0.12	kWh/kWh	Projection from WEO NZ2050, industry - metals & concrete<br>Energy intensity from Hertwich et al. (2015)
**Agriculture**	*Total*	EP	1,068	1,015	949	886	851	819	789	MJ/cap	-
**Freight**	Road (direct)	EP	2.34	2.19	1.96	1.75	1.60	1.49	1.40	MJ/tkm	-
	Rail (direct)	EP	0.17	0.16	0.15	0.14	0.14	0.13	0.13	MJ/tkm	-
	Road (beyond direct)	EP-EX	19%	19%	20%	20%	20%	20%	18%	-	Indirect domestic fraction of sector's energy footprint
	Rail (beyond direct)	EP-EX	52%	52%	51%	50%	47%	44%	39%	-	Indirect domestic of sector's energy footprint
Imported energy											
**Residential**	Clothing	EX-HH	3,933	3,740	3,557	3,383	3,217	3,059	2,909	MJ/cap	Projection from Millward-Hopkins et al. (2023)
	Cleaning products*	EX-HH	4,550	4,409	4,268	4,126	4,028	3,981	3,934	MJ/cap	Projection from WEO NZ2050, industry - chemicals
	Household furnishings*		19	18	17	16	15	15	14	MJ/m^2	Projection from WEO NZ2050, industry - all<br>Energy intensity from Hoxha & Jusselme (2017)
	Other consumer goods	EX-HH	513	491	470	448	425	405	385	MJ/cap	Projection from WEO NZ2050, industry - all
**Transport**	Road transport (beyond direct)	EX-Tot	0.76	0.71	0.66	0.58	0.49	0.42	0.34	MJ/pkm	Projection from WEO NZ2050, industry - metals
	Rail transport (beyond direct)	EX-Tot	0.50	0.46	0.43	0.38	0.32	0.27	0.22	MJ/pkm	Projection from WEO NZ2050, industry - metals
	Air transport (beyond direct)	EX-Tot	0.44	0.42	0.40	0.38	0.35	0.33	0.31	MJ/pkm	Projection from WEO NZ2050, industry - metals & concrete
**Services**	Healthcare	EP-EX	60%	60%	60%	60%	60%	60%	60%	-	Imported fraction of sector's energy footprint
	Education	EP-EX	42%	42%	42%	42%	42%	42%	42%	-	Imported fraction of sector's energy footprint
	Telecommunication	EP-EX	70%	70%	70%	70%	70%	70%	70%	-	Imported fraction of sector's energy footprint
	Public space*	EP-EX	45%	45%	45%	45%	45%	45%	45%	-	Imported fraction of sector's energy footprint
	Public administration & research*	EP-EX	40%	40%	40%	40%	40%	40%	40%	-	Imported fraction of sector's energy footprint
	Trade	EP-EX	73%	73%	73%	73%	73%	73%	73%	-	Imported fraction of sector's energy footprint
**Industry**	Water supply & waste management	EP-EX	11%	11%	11%	11%	11%	11%	11%	-	Imported fraction of sector's energy footprint
	Construction	EP-EX	66%	66%	66%	66%	66%	66%	66%	-	Imported fraction of sector's energy footprint
	Electricity infrastructure		0.016	0.015	0.014	0.013	0.013	0.012	0.011	kWh/kWh	Projection from WEO NZ2050, industry - metals & concrete<br>Energy intensity from Hertwich et al. (2015)
**Agriculture**	*Total*	EP-EX	68%	68%	68%	68%	68%	68%	68%	-	Imported fraction of sector's energy footprint
**Freight**	Road (beyond direct)	EP-EX	55%	55%	57%	57%	57%	56%	54%	-	Imported fraction of sector's energy footprint
	Rail (beyond direct)	EP-EX	63%	63%	62%	61%	58%	55%	50%	-	Imported fraction of sector's energy footprint

Supplementary References

1) Food and Agriculture Organisation of the United Nations (FAO). *Selected indicators for Switzerland*. www.fao.org/faostat/en/#country/211 (accessed 04/03/2025)

2) Willett et al. Food in the Anthropocene: the EAT–Lancet Commission on healthy diets from sustainable food systems. *The Lancet* **393**, 447-492 (2019).

3) Millward-Hopkins J, Steinberger JK, Rao ND & Oswald Y. Providing Decent Living with Minimum Energy: A Global Scenario. *Global Environmental Change* **65**, 102168 (2020). doi.org/10.1016/j.gloenvcha.2020.102168

4) Federal statistical office (FSO). *Floor space per occupant: Buildings and dwellings statistics* (Federal statistical office, Section Population, Switzerland). www.bfs.admin.ch/bfs/en/home/statistics/construction-housing/dwellings/housing-conditions/floor-area-person.html (accessed 04.03.2025)

5) Federal statistical office (FSO). *Average living space by nationality of household members: Buildings and dwellings statistics* (Federal statistical office, Section Population, Switzerland). www.bfs.admin.ch/bfs/en/home/statistics/construction-housing/dwellings/housing-conditions/floor-area-person.assetdetail.27585711.html (accessed 04.03.2025)

6) Gleick PH. Basic Water Requirements for Human Activities: Meeting Basic Needs. *Water International* **21**, 83-92 (1996).

7) United Nations. *Global Issues: Water*, https://www.un.org/en/global-issues/water (accessed 03/03/2025)

8) Eurostat. *Water statistics: Household water use*, https://ec.europa.eu/eurostat/statistics-explained/index.php?title=Water_statistics (accessed 04/03/2025)

9) Eurostat. *Sustainable Development goals indicators: raw material consumption* https://ec.europa.eu/eurostat/databrowser/view/sdg_12_21/default/table (accessed 04/03/2025)

10) Vélez-Henao JA & Pauliuk S. Material Requirements of Decent Living Standards. *Environmental science & technology* **57**, 14206-14217 (2023). https://doi.org/10.1021/acs.est.3c03957

11) Kepios, *Digital 2023 Switzerland*, https://datareportal.com/reports/digital-2023-switzerland (accessed 04/03/2025)

12) Rao ND, Min J & Mastrucci A. Energy requirements for decent living in India, Brazil and South Africa. *Nature Energy* **4**, 1025-1032 (2019). https://doi.org/10.1038/s41560-019-0497-9

13) Millward-Hopkins J. Inequality can double the energy required to secure universal decent living. *Nature Communications*, **13**, 5028 (2022). https://doi.org/10.1038/s41467-022-32729-8

14) FSO (2015), Population's travel behaviour 2015, Federal Statistical Office (FSO), document number: 1697-1500, https://www.are.admin.ch/dam/are/en/dokumente/verkehr/dokumente/mikrozensus/verkehrsverhalten-der-bevolkerung-2015.pdf.download.pdf/ (accessed 104/03/2025)

15) Kallis G, Kalush M, O'Flynn, H Rossiter J, Ashford N. "Friday off": Reducing Working Hours in Europe. *Sustainability* **5**, 1545-1567 (2013). https://doi.org/10.3390/su5041545

16) Federal statistical office (FSO). *Actual hours worked: Key figures, 2023* (Federal statistical office, Section Labour Force, Switzerland). https://www.bfs.admin.ch/bfs/en/home/statistics/work-income/employment-working-hours/working-hours/actual-hours-worked.html (accessed 04.03.2025)

17) Federal statistical office (FSO). *Persons employed part-time and full-time, Job-sharing* (Federal statistical office, Section Labour Force, Switzerland). https://www.bfs.admin.ch/bfs/en/home/statistics/work-income/employment-working-hours/working-hours/actual-hours-worked.html (accessed 04.03.2025)

18) Kompil M, Jacobs-Crisioni C, Dijkstra L & Lavalle C. Mapping accessibility to generic services in Europe: A market-potential based approach. *Sustainable Cities and Society* **47**, 101372 (2019). https://doi.org/10.1016/j.scs.2018.11.047

19) Federal statistical office (FSO). *Accessibility: Services to the population* (Federal statistical office, Europe Area, Switzerland). https://www.bfs.admin.ch/bfs/fr/home/statistiques/themes-transversaux/analyses-spatiales/services-population/accessibilite.html (accessed 04.03.2025)

20) Citec Ingénieurs SA (2021). *Perspectives pour augmenter la part modale des transports publics: Plus d'agilité pour préparer le futur* (Public Transport Union, Switzerland, Bern). https://www.voev.ch/fr/Services/Publications/Ecrits-UTP (accessed 04.03.2025)

21) Grubler et al. A low energy demand scenario for meeting the 1.5 °C target and sustainable development goals without negative emission technologies. *Nature Energy* **3**, 515-527 (2018). https://doi.org/10.1038/s41560-018-0172-6

22) Rao ND & Min J. Decent Living Standards: Material Prerequisites for Human Wellbeing. *Social Indicators Research* **138**, 225-244 (2018). https://doi.org/10.1007/s11205-017-1650-0

23) World Bank, *Worldwide Governance Indicators: World Bank Open Data,* https://databank.worldbank.org/source/worldwide-governance-indicators/ (accessed 04/03/2025)

24) International Energy Agency. *World energy outlook 2023* (Paris, France: International Energy Agency 2023) https://www.iea.org/reports/world-energy-outlook-2023 (accessed 10/05/2024)

25) Millward-Hopkins J, Purnell P & Baurley S. Scenarios for reducing the environmental impacts of the UK clothing economy. *Journal of Cleaner Production* **420**, 138352 (2023).

26) Hertwich et al. Integrated life-cycle assessment of electricity-supply scenarios confirms global environmental benefit of low-carbon technologies. *Proceedings of the National Academy of Sciences*, **112**, 6277-6282 (2015).

27) Hoxha E & Jusselme T. On the necessity of improving the environmental impacts of furniture and appliances in net-zero energy buildings. *Science of the Total Environment* **596-597**, 405-416 (2017).

28) Panos, E., Kannan, R., Hirschberg, S., & Kober, T. An assessment of energy system transformation pathways to achieve net-zero carbon dioxide emissions in Switzerland. *Communications Earth & Environment*, **4**, 157 (2023).
