# Supplementary material for: Energy requirements for securing wellbeing in Switzerland and the space for affluence and inequality
Source: Nat Commun. 2025 Apr 30;16:4066. doi: 10.1038/s41467-025-59276-2 (PMC12043928; doi:10.1038/s41467-025-59276-2)
Supplement: Supplementary file 2 — Description of addtional supplementary file [file 41467_2025_59276_MOESM2_ESM.pdf]

# Description of Additional Supplementary File

**Description of Supplementary Data 1 -** This includes all critical input and output data of the DLE model.
